# Analysis of lymph node recurrence patterns and risk factors in locally advanced esophageal carcinoma following neoadjuvant therapy

**DOI:** 10.3389/fonc.2025.1668649

**Published:** 2025-10-14

**Authors:** Yingxin Liu, Xiaojun Wang, Zixuan Ni, Qiang Zeng, Yutao Li, Puchang Zhang, Pudong Qian, Yatian Liu

**Affiliations:** ^1^ Department of Radiation Oncology, Nanjing Medical University Affiliated Cancer Hospital, Jiangsu Province Cancer Hospital, Jiangsu Provincial Cancer Prevention and Treatment Research Institute, Nanjing, Jiangsu, China; ^2^ Department of Thoracic Surgery, Nanjing Medical University Affiliated Cancer Hospital, Jiangsu Province Cancer Hospital, Jiangsu Provincial Cancer Prevention and Treatment Research Institute, Nanjing, Jiangsu, China

**Keywords:** esophageal cancer, neoadjuvant therapy, adjuvant therapy, lymph nodes recurrence, risk factors

## Abstract

**Background:**

Although neoadjuvant therapy followed by surgery is the standard treatment for resectable locally advanced esophageal carcinoma, heterogeneity exists in neoadjuvant regimens within real-world practice. This study characterizes lymph node recurrence (LNR) patterns and compares LNR risk factors across different neoadjuvant approaches to better elucidate therapeutic efficacy.

**Methods:**

Data were collected from esophageal carcinoma (EC) patients who underwent surgery following neoadjuvant therapy between January 2018 and December 2023. Neoadjuvant regimens included neoadjuvant chemotherapy (NCT), neoadjuvant chemoimmunotherapy (NICT), and neoadjuvant chemoradiotherapy (NCRT)/neoadjuvant chemoradiotherapy combined with immunotherapy (NCRT/NICRT). Regional lymph node stations were defined per the Japanese Esophageal Society (JES) criteria. Cox proportional hazards models were used to identify factors associated with LNR.

**Results:**

This study enrolled 658 patients, including 195 with postoperative LNR. Among all EC cases, the most frequent recurrence sites were No. 106tb (5.5%), No. 109 (5.5%), and No. 104 (8.7%). The most frequent recurrence sites in the NCT group were No. 104 (10.0%) and No. 106tb (7.9%); in the NICT group, No. 104 (8.2%) was the most common; in the NCRT/NICRT group, No. 101 (7.0%) and No. 104 (7.0%) demonstrated the highest recurrence rates. In upper thoracic EC, No. 101, No. 104, and No. 105 were common recurrence sites with metastasis rates exceeding 5%; in mid-thoracic EC, No. 104 (11.9%) showed the highest recurrence frequency; in lower thoracic and gastroesophageal junction EC, No. 104 (5.5%) and No. 16 (4.8%) were frequent recurrence locations. Lymph node dissection count and ypT and/or ypN stage correlated with recurrence risk across neoadjuvant regimens. Adjuvant radiotherapy demonstrated lower lymph node metastasis rates in the tracheoesophageal groove and upper mediastinal LNR.

**Conclusions:**

The tracheoesophageal groove and supraclavicular lymph nodes represent common recurrence sites in neoadjuvant therapy and upper thoracic EC. Lower thoracic and gastroesophageal junction EC require vigilance for the risk of supraclavicular nodal metastasis. Lymph node yield and T/N stages correlate with recurrence risk.

## Introduction

Esophageal carcinoma (EC) ranks as the seventh most common malignancy globally and the sixth leading cause of cancer-related mortality, with incidence demonstrating a sustained upward trajectory (1). China represents a high-incidence region where EC exhibits distinctive epidemiological patterns—sixth in incidence and fifth in mortality among all malignancies—with squamous cell carcinoma (SCC) constituting the predominant histological subtype (2, 3). The probability of lymph node metastasis significantly correlates with advancing T stage, leading to the clinical designation of cT1b-2N+M0 or cT3-4NanyM0 disease as locally advanced esophageal cancer (LAEC). While surgical resection remains the cornerstone of management for resectable LAEC, the intricate lymphatic architecture of the esophagus presents unique oncological challenges. The thoracic esophageal lymphatic network establishes multidirectional drainage pathways connecting cervical, mediastinal, and abdominal nodal basins (4). This anatomical characteristic underlies EC’s propensity for early nodal dissemination and contributes to suboptimal disease control, with postoperative recurrence rates exceeding 40% following surgery alone.

Advances in multidisciplinary management have demonstrated significant survival improvements through combined modality approaches incorporating surgery, chemotherapy, immunotherapy, and radiotherapy. Consequently, neoadjuvant chemotherapy (NCT) and neoadjuvant chemoradiotherapy (NCRT) have been established as standard therapeutic regimens for LAEC. Nevertheless, approximately 50% of patients experience disease progression during long-term follow-up (5–7). The advent of immunotherapy has introduced neoadjuvant chemoimmunotherapy (NICT) as a potentially superior alternative for LAEC patients (8–11), although large-scale trials confirming its advantage over NCRT remain pending. Pathological staging (ypTNM) serves as a critical prognostic tool, with tumor invasion depth and lymph node metastasis burden being key determinants of survival in neoadjuvant-treated esophageal squamous cell carcinoma (ESCC) (12).

A large retrospective cohort study demonstrated that supraclavicular and upper mediastinal lymph nodes are common recurrence sites in postoperative ESCC patients, with neoadjuvant therapy only reducing recurrence rates in these specific nodal regions compared to surgery alone (13). A single-arm phase II clinical trial from our institution revealed that among 47 patients receiving NICT followed by surgery, locoregional recurrence approached 30% (9); Tian et al. analyzed lymph node metastasis patterns across NCT, NICT, and NCRT regimens, showing significantly reduced metastasis rates at No. 101R and No. 106rec in the NCRT cohort (12). To date, most studies have remained focused on the relationship between pathological factors and survival in EC after neoadjuvant therapy. Research on lymph node recurrence (LNR) patterns in these patients remains limited. It remains unclear whether LNR sites differ among EC patients with distinct primary tumor locations compared to those treated with surgery alone, or how variations in neoadjuvant regimens and the application of adjuvant therapies influence lymph node metastasis patterns.

This study analyzed LNR patterns in EC patients with distinct primary tumor locations, explored the characteristics of postoperative LNR patterns in LAEC patients receiving different neoadjuvant therapies, compared differences among treatment strategies, and determined recurrence-predilection sites under specific therapeutic modalities to provide evidence-based foundations for individualized recurrence prevention strategies.

## Patients and materials

**Figure 1:**
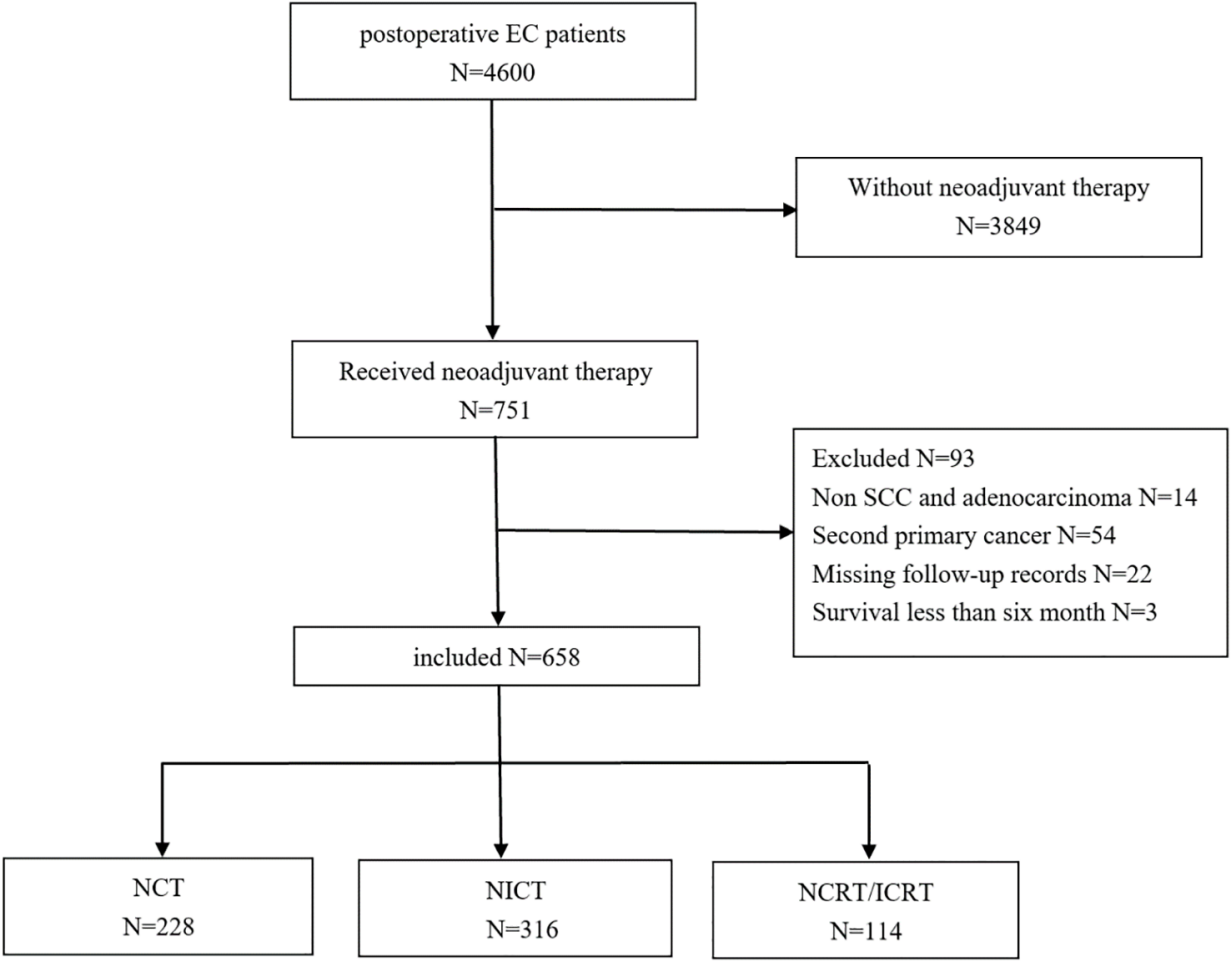
Flowchart for the patient selection.

This retrospective analysis received approval from the Medical Ethics Committee (KY-2024-108) with waiver of informed consent. EC patients who underwent neoadjuvant therapy followed by esophagectomy at Jiangsu Cancer Hospital from January 2018 to December 2023 were enrolled (**Figure 1**). Distant organ metastases were excluded preoperatively using chest and abdominal computed tomography (CT) or positron emission tomography (PET). Inclusion criteria were as follows: 1) age ≥ 18 years; 2) EC patients treated with NCT, NICT, or NCRT/neoadjuvant chemoradiotherapy combined with immunotherapy (NICRT); 3) SCC or adenocarcinoma; and 4) lymph node recurrence occurring >6 months post-surgery. Exclusion criteria were as follows: 1) cervical EC; 2) history of other malignant tumors; 3) macroscopically residual tumor (R2 resection); 4) incomplete medical records or follow-up documentation; and 5) postoperative survival <6 months.

All patients completed at least one cycle of neoadjuvant therapy prior to surgery. The neoadjuvant and adjuvant chemotherapy regimens consisted of taxane-platinum doublet-based therapy, with some neoadjuvant protocols incorporating additional S-1 or fluorouracil. Adjuvant radiotherapy was delivered at a median total dose of 42.8 Gy (range, 40.0–50.4 Gy) in conventional fractionation (1.8–2.0 Gy per fraction, five fractions per week). The clinical target volume included bilateral supraclavicular regions, upper mediastinal nodal drainage areas, and the postoperative tumor bed. In selected cases, coverage was extended to encompass high-risk regions such as the middle mediastinum and the left gastric nodal basin.

### Surgical procedures

All patients underwent radical esophagectomy with lymphadenectomy after confirming disease non-progression via contrast-enhanced CT scans of the neck, chest, and abdomen following neoadjuvant therapy completion. The majority of patients underwent minimally invasive esophagectomy, primarily via the Ivor–Lewis or McKeown approach, while a minority received open procedures. Surgical approaches were selected by thoracic surgeons based on the neoadjuvant regimen and imaging. Standard mediastinal lymphadenectomy included recurrent laryngeal nerve stations with two-field dissection. An extended two-field or three-field dissection was implemented for suspected cervical nodal involvement. The surgical approach was determined by thoracic surgeons based on the neoadjuvant regimen and imaging findings. Patients underwent mediastinal lymphadenectomy, including recurrent laryngeal nerve nodal stations, with routine two-field dissection performed. When surgeons suspected cervical lymph node positivity, an extended two-field or three-field lymphadenectomy was conducted.

### Histological response criteria

Pathological response was assessed by two experienced pathologists by measuring the percentage of residual viable tumor in the resected primary tumor. Pathological examination included tumor type, grade, resection margins, number of involved lymph nodes, and tumor staging. Pathological staging followed the 8th edition of the TNM classification by the American Joint Committee on Cancer (AJCC) (14). Complete regression of both primary tumor and metastatic lymph nodes was defined as pathological complete response (pCR) (15). Tumor regression grade (TRG) was classified according to the AJCC 8th edition of the TNM staging criteria, as follows: no residual cancer cells (TRG0), 1%–10% residual cancer cells (TRG1), 11%–50% residual cancer cells (TRG2), and >50% residual cancer cells (TRG3) (14).

### Definition of lymph node recurrence and region

Postoperative follow-up included contrast-enhanced CT scans of the neck, chest, and abdomen every 3 months during the first 2 years after surgery and every 6 months from years 3 to 5. Imaging evaluations were performed independently by two experienced radiologists, with discrepancies resolved by consensus. Recurrence was defined as progression ≥6 months postoperatively confirmed by CT/PET. Local recurrence referred to anastomotic recurrence and/or cervical, mediastinal, or abdominal lymph node metastasis. LNR was radiologically or pathologically defined by the presence of one or more of the following features: clustered or morphologically suspicious nodes on CT, central liquefactive necrosis, short-axis diameter >10 mm (>5 mm for upper mediastinal nodes), significantly elevated metabolic uptake on PET–CT compared to background tissue, or positive cytological confirmation via fine-needle aspiration. Anastomotic recurrence was diagnosed based on postoperative endoscopic biopsy pathology, supported by contrast-enhanced CT findings showing irregular anastomotic thickening >5 mm with heterogeneous enhancement, or focally increased uptake on PET–CT. The lymph node metastasis rate was calculated as follows: (number of cases with imaging or pathologically confirmed metastatic involvement at a given nodal station/total number of cases) × 100%.

The nomenclature of regional lymph nodes in EC follows the Japanese Esophageal Society (JES) definitions (16). In this study, esophageal lymph node stations were categorized into 1) cervical lymph nodes including No. 101, No. 102, No. 103, and No. 104; 2) upper mediastinal lymph nodes including No. 105, No. 106Rec, No. 106tb, and No. 106pre; 3) middle mediastinal lymph nodes including No. 107, No. 108, and No. 109; 4) lower mediastinal lymph nodes including No. 110, No. 111, No. 112, No. 113, and No. 114; and 5) abdominal lymph nodes mainly including perigastric nodes (Nos. 1–3), left gastric artery nodes (No. 7), celiac nodes (No. 9), para-aortic nodes (No. 16), and other abdominal lymph nodes.

### Endpoints

The primary objective was recurrence rates and sites of regional lymph nodes (per JES nomenclature), with secondary analysis of LNR risk factors across treatment modalities.

Continuous variables were described using median and range, and categorical variables were summarized as frequencies and percentages. Due to limited cases of ypTis stage, ypT0 and ypTis were combined. Associations between clinicopathological factors and LNR across various neoadjuvant treatment regimens were examined using the chi-square test. Multivariable regression analysis of survival outcomes included covariates such as age, sex, ypT, ypN, surgical method, and postoperative adjuvant therapy. The Schoenfeld residual method was used to test whether all covariates met the proportional hazards assumption. Multivariable Cox proportional hazards regression models were used to assess the association between recurrence-free survival (RFS) with different neoadjuvant therapy types and patient characteristics, including age, pathological type, TRG, and number of lymph nodes dissected. Cumulative incidence curves were generated to visualize the probability of LNR over time. Statistical significance was set at *p* < 0.05.

## Results

### Patient characteristics

The study enrolled 658 EC patients who underwent surgery after neoadjuvant therapy, including 640 squamous cell carcinoma patients (97.3%) and 18 adenocarcinoma patients (2.7%). The cohort exhibited a male-to-female ratio of 4.7:1. Patients with ≥15 mediastinal lymph nodes dissected accounted for 52.4%. Neoadjuvant regimens included the following: NCT in 228 patients, NICT in 316 patients, and NCRT/NICRT in 114 patients. The median radiotherapy dose delivered was 31.3 Gy (range, 30.0–41.4 Gy), administered in daily fractions of 1.8–2.5 Gy, five times per week. The median follow-up time was 41 months (95% CI, 37.649–44.351). Detailed baseline characteristics are presented in **Table 1**.

**Table 1:** Clinical characteristics of the general population and different neoadjuvant treatment schemes.

Characteristics	Total (N = 658)	NCT (N = 228)	NICT (N = 316)	NCRT/NICRT (N = 114)	*p*
Sex					0.313
Male	544 (82.7)	182 (79.8)	264 (83.5)	98 (85.9)	
Female	114 (17.3)	46 (20.2)	52 (16.5)	16 (14.1)	
Age (years)					0.088
<65	376 (57.1)	124 (54.4)	194 (61.4)	58 (50.9)	
≥65	282 (42.9)	104 (45.6)	122 (38.6)	56 (49.1)	
Pathological type					0.026
Squamous cell carcinoma	640 (97.3)	217 (95.2)	309 (97.8)	114 (100.0)	
Adenocarcinoma	18 (2.7)	11 (4.8)	7 (2.2)	0 (0.0)	
Location					0.003
Upper thoracic	75 (11.4)	37 (16.2)	30 (9.5)	8 (7.0)	
Mid thoracic	310 (47.1)	94 (41.2)	151 (47.8)	65 (57.0)	
Lower thoracic	237 (36.0)	78 (34.2)	119 (37.6)	40 (35.1)	
Gastroesophageal junction	36 (5.5)	19 (8.4)	16 (5.1)	1 (0.9)	
Pathological differentiation					<0.001
No carcinoma	202 (30.7)	38 (16.7)	97 (30.6)	67 (58.8)	
Well differentiated	36 (5.5)	18 (7.9)	15 (4.8)	3 (2.6)	
Moderately differentiated	324 (49.2)	127 (55.7)	162 (51.3)	35 (30.7)	
Poorly differentiated	96 (14.6)	45 (19.7)	42 (13.3)	9 (7.9)	
Tumor regression grade (TRG)					<0.001
0	181 (27.5)	33 (14.4)	86 (27.2)	62 (54.4)	
1	152 (23.1)	53 (23.3)	76 (24.0)	23 (20.2)	
2	214 (32.5)	83 (36.4)	107 (33.9)	24 (21.0)	
3	111 (16.9)	59 (25.9)	47 (14.9)	5 (4.4)	
Total lymph nodes removed					0.002
<15	313 (47.6)	128 (56.1)	143 (45.3)	42 (36.8)	
≥15	345 (52.4)	100 (43.9)	173 (54.7)	72 (63.2)	
ypT					<0.001
T0/Tis	205 (31.2)	38 (16.7)	100 (31.7)	67 (58.8)	
T1	116 (17.6)	41 (17.9)	62 (19.6)	13 (11.4)	
T2	123 (18.7)	50 (21.9)	56 (17.7)	17 (14.9)	
T3	201 (30.5)	92 (40.4)	92 (29.1)	17 (14.9)	
T4	13 (2.0)	7 (3.1)	6 (1.9)	0 (0.0)	
ypN					0.54
N0	418 (63.5)	134 (58.8)	206 (65.2)	78 (68.4)	
N1	161 (24.5)	60 (26.3)	75 (23.7)	26 (22.8)	
N2	58 (8.8)	26 (11.4)	25 (7.9)	7 (6.2)	
N3	21 (3.2)	8 (3.5)	10 (3.2)	3 (2.6)	
Surgical approach					<0.001
Ivor–Lewis	371 (56.4)	133 (58.3)	189 (59.8)	49 (42.9)	
McKeown	232 (35.3)	60 (26.3)	109 (34.5)	63 (55.3)	
Sweet	31 (4.7)	21 (9.3)	10 (3.2)	0 (0.00)	
Other	24 (3.6)	14 (6.1)	8 (2.5)	2 (1.8)	
Anastomosis site					<0.001
Cervical	253 (38.4)	73 (32.0)	115 (36.4)	65 (57.0)	
Upper thoracic	368 (56.0)	137 (60.1)	182 (57.6)	49 (43.0)	
Other	37 (5.6)	18 (7.9)	19 (6.0)	0 (0.00)	
Adjuvant therapy					<0.001
No therapy	259 (39.4)	78 (34.2)	115 (36.4)	44 (38.6)	
Without radiotherapy	237 (36.0)	75 (32.9)	82 (25.9)	5 (4.4)	
Including radiotherapy	162 (24.6)	75 (32.9)	119 (37.7)	65 (57.0)	

NCT, neoadjuvant chemotherapy; NICT, neoadjuvant chemoimmunotherapy; NCRT, neoadjuvant chemoradiotherapy; NICRT, neoadjuvant chemoradiotherapy plus immunotherapy.

### Patterns of LNR locations

**Figure 2:**
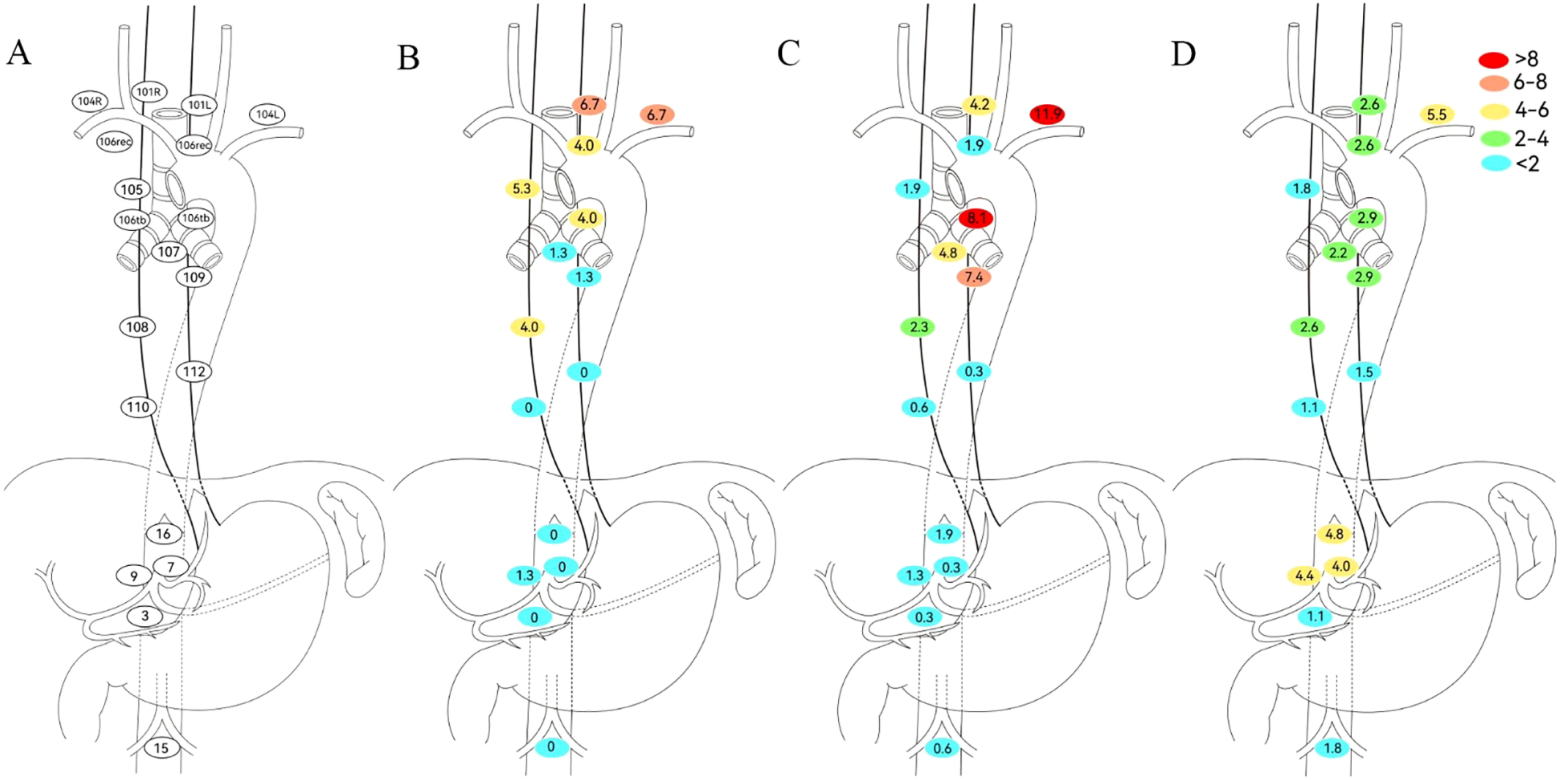
Defining regional lymph node recurrence based on primary tumor location. **(A)** Japanese Esophageal Society (JES) reference diagram. **(B)** Upper thoracic. **(C)** Middle thoracic. **(D)** Lower thoracic. No. 101, cervical paraesophageal lymph nodes; No. 104, supraclavicular lymph nodes; No. 105, upper thoracic paraesophageal lymph nodes; No. 106rec, recurrent nerve lymph nodes; No. 106tb, tracheobronchial lymph nodes; No. 107, subcarinal lymph nodes; No. 108, middle thoracic paraesophageal lymph nodes; No. 109, main bronchus lymph nodes; No. 110, lower thoracic paraesophageal lymph nodes; No. 112, posterior mediastinal lymph nodes; No. 3, lymph nodes along the lesser curvature of the stomach; No. 7, lymph nodes along the left gastric artery; No. 9, lymph nodes along the celiac artery; No. 15, lymph nodes along the middle colic artery; No. 16, lymph nodes around the abdominal aorta.

This study consolidated bilateral symmetric lymph node stations into single regions. Given the small number of pure gastroesophageal junction (GEJ) tumors and their shared drainage patterns with lower thoracic esophageal cancers, lower thoracic and GEJ EC patients were combined for analysis. Site-specific LNR rates based on primary tumor location are detailed in **Figure 2**. In upper thoracic EC, No. 101, No. 104, and No. 105 were common recurrence sites with metastasis rates of 6.7%, 6.7%, and 5.3%, respectively, while abdominal LNR was rare. For mid-thoracic EC, No. 104 (11.9%) was the predominant recurrence site, with No. 106tb (8.1%) and No. 109 (7.4%) also meriting attention. Notably, No. 104 showed the highest LNR rate (>5%) in lower thoracic/GEJ EC, followed by No. 16 (4.8%), No. 9 (4.4%), and No. 7 (4.0%). The detailed number of lymph node metastases is provided in **Table 2**.

**Table 2:** Lymph node metastasis count stratified by neoadjuvant therapy regimen and tumor location.

Lymph node location	Upper thoracic	Mid thoracic	Lower thoracic/GEJ	*p*	NCT	NICT	NCRT/NICRT	*p*
101L	2 (18.2)	6 (54.5)	3 (27.3)	0.415	1 (9.1)	4 (36.4)	6 (54.5)	0.007
101R	3 (21.4)	7 (50.0)	4 (28.6)	0.394	4 (28.6)	8 (57.1)	2 (14.3)	0.935
104L	4 (11.8)	22 (64.7)	8 (23.5)	0.076	15 (44.1)	15 (44.1)	4 (11.8)	0.431
104R	1 (4.2)	16 (66.7)	7 (29.1)	0.13	8 (33.3)	12 (50.0)	4 (16.7)	0.981
105	4 (26.7)	6 (40.0)	5 (33.3)	0.169	5 (33.3)	8 (53.3)	2 (13.4)	0.887
106tbL	3 (10.0)	20 (66.7)	7 (23.3)	0.078	14 (46.7)	13 (43.3)	3 (10.0)	0.297
106tbR	0 (0.0)	5 (83.3)	1 (16.7)	0.321	4 (66.7)	2 (33.3)	0 (0.0)	0.279
106recL	1 (16.7)	5 (83.3)	0 (0.0)	0.077	4 (66.7)	2 (33.3)	0 (0.0)	0.279
106recR	2 (20.0)	3 (30.0)	5 (50.0)	0.334	3 (30.0)	6 (60.0)	1 (10.0)	0.837
107	1 (4.6)	14 (63.6)	7 (31.8)	0.25	10 (45.5)	11 (50.0)	1 (4.5)	0.231
108	3 (17.6)	7 (41.2)	7 (41.2)	0.695	5 (29.4)	8 (47.1)	4 (23.5)	0.767
109L	1 (5.2)	12 (63.2)	6 (31.6)	0.336	7 (36.8)	11 (57.9)	1 (5.3)	0.356
109R	0 (0.0)	14 (82.4)	3 (17.6)	0.011	5 (29.4)	9 (52.9)	3 (17.7)	0.893
110	0 (0.0)	3 (50.0)	3 (50.0)	1	3 (50.0)	3 (50.0)	0 (0.0)	0.568
112	0 (0.0)	1 (20.0)	4 (80.0)	0.251	1 (20.0)	4 (80.0)	0 (0.0)	0.492
3	0 (0.0)	1 (25.0)	3 (75.0)	0.597	2 (50.0)	2 (50.0)	0 (0.0)	0.833
7	0 (0.0)	2 (16.7)	10 (83.3)	0.018	8 (66.7)	3 (25.0)	1 (8.3)	0.081
9	1 (5.9)	4 (23.5)	12 (70.6)	0.048	6 (35.3)	6 (35.3)	5 (29.4)	0.357
15	0 (0.0)	2 (28.6)	5 (71.4)	0.382	5 (71.4)	2 (28.6)	0 (0.0)	0.157
16	0 (0.0)	6 (33.4)	12 (66.6)	0.058	6 (33.3)	8 (44.5)	4 (22.2)	0.854

GEJ, gastroesophageal junction; NCT, neoadjuvant chemotherapy; NICT, neoadjuvant chemoimmunotherapy; NCRT, neoadjuvant chemoradiotherapy; NICRT, neoadjuvant chemoradiotherapy plus immunotherapy.

**Figure 3:**
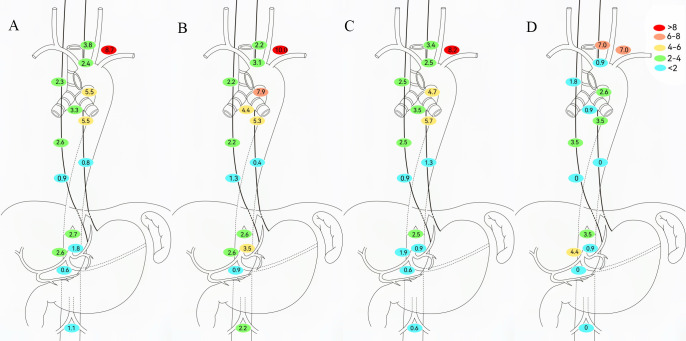
Determining regional lymph node recurrence patterns based on neoadjuvant therapy modalities. **(A)** All patients. **(B)** Neoadjuvant chemotherapy (NCT). **(C)** Neoadjuvant chemoimmunotherapy (NICT). **(D)** Neoadjuvant chemoradiotherapy (NCRT)/neoadjuvant chemoradiotherapy plus immunotherapy (NCRT/NICRT).

Among the 658 patients, the anastomotic recurrence rate was 3.0%; No. 104, No. 106tb, and No. 109 were the most frequent LNR sites (**Figure 3**). Given that radiotherapy enhances local control, LNR patterns were analyzed by neoadjuvant therapy type (**Figure 3**). The NCT and NICT groups showed the highest recurrence at station 104 (10.0% and 8.2%, respectively). NICT recipients had lower 106tb LNR rates versus NCT (4.7% vs 7.9%). No significant differences in LNR rates were observed at other mediastinal or abdominal stations between these regimens. The NCRT/NICRT group had significantly reduced mediastinal LNR; however, No. 101 and No. 104 showed recurrence rates exceeding 5% (7.0% and 7.0%, respectively), followed by No. 9 (4.4%) and No. 16 (3.5%).

**Figure 4:**
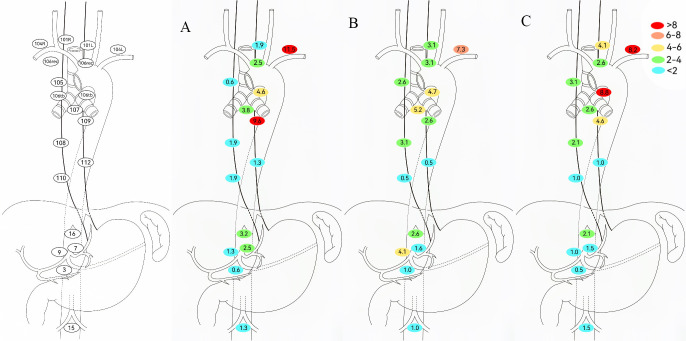
Determination of regional lymph node recurrence patterns based on adjuvant therapy modalities. **(A)** Adjuvant including radiotherapy. **(B)** Adjuvant without radiotherapy. **(C)** No adjuvant therapy.

To evaluate adjuvant therapy’s impact on LNR in EC patients, this study excluded NCRT/NICRT cases to minimize confounding from their local control effects and analyzed LNR patterns in NCT/NICT patients (**Figure 4**). Among patients without adjuvant therapy, metastasis rates exceeded 5% at No. 104 (8.2%) and No. 106tb (8.8%). Patients receiving adjuvant chemotherapy/chemoimmunotherapy showed reduced metastasis trends in these regions. Adjuvant radiotherapy yielded lower tracheoesophageal groove/upper mediastinal LNR rates than other groups, but required vigilance for No. 104 (11.5%) and No. 109 (9.6%).

### Factors associated with LNR in EC patients receiving distinct neoadjuvant therapy

**Figure 5:**
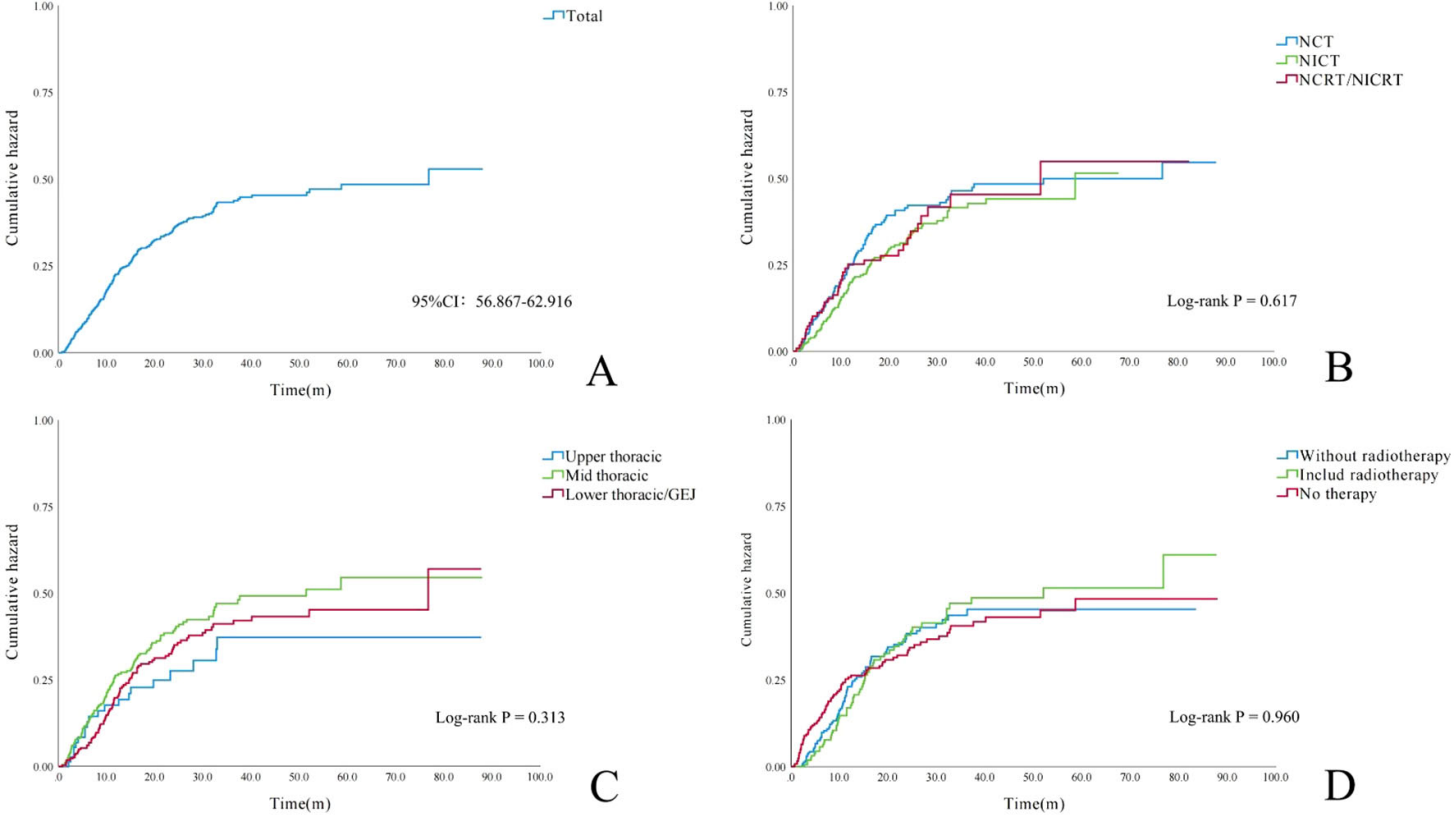
Cumulative incidence curves of lymph node metastasis. **(A)** Entire cohort (N = 658 patients). **(B)** By neoadjuvant therapy regimen (*p* = 0.617). **(C)** By primary tumor location (*p* = 0.313). **(D)** By adjuvant treatment strategy (*p* = 0.960).

The median time to LNR was 25.7 months (95% CI, 28.54–31.74). As shown in **Figure 5**, the cumulative risk of LNR increased over time across all treatment groups and tumor locations. In the short term, the NCT group, mid-thoracic tumor group, and untreated group exhibited slightly higher cumulative recurrence risks; however, these differences did not significantly influence the overall trend.

Cox regression analysis (**Table 3**) indicated that among all esophageal cancer patients, higher ypT and ypN stages were associated with an increased risk of LNR. Furthermore, a lower total number of dissected lymph nodes was correlated with a higher LNR risk. The ypN stage was identified as an independent risk factor for LNR across all neoadjuvant treatment regimens. In the NCT subgroup, receipt of adjuvant therapy was significantly associated with LNR. Among patients who received NICT, histological type, ypT stage, total number of dissected lymph nodes, and anastomotic site were significantly associated with LNR. For those undergoing NCRT/NICRT, the total number of dissected lymph nodes was significantly associated with LNR.

**Table 3:** Multivariate analysis of clinicopathological factors for recurrence-free survival.

Factor	Total		NCT		NICT		NCRT/NICRT	
	HR (95% CI)	*p*	HR (95% CI)	*p*	HR (95% CI)	*p*	HR (95% CI)	*p*
Sex	1.37 (0.91–2.05)	0.131	1.62 (0.85–3.07)	0.14	1.35 (0.72–2.54)	0.347	0.49 (0.14–1.82)	0.293
Age	0.96 (0.72–1.28)	0.79	1.14 (0.69–1.87)	0.595	1.11 (0.69–1.75)	0.665	0.53 (0.23–1.19)	0.123
Pathological type	1.79 (0.68–4.73)	0.238	0.87 (0.26–2.86)	0.816	16.86 (1.93–147.03)	0.011	—	—
Location	1.25 (0.77–2.05)	0.136	0.55 (0.15–2.07)	0.118	1.08 (0.47–2.49)	0.937	1.12 (0.22–5.69)	0.449
Pathological differentiation	2.73 (0.47–15.69)	0.279	2.59 (0.02–354.98)	0.932	2.64 (0.29–23.33)	0.673	2.86 (0.40–20.42)	0.277
Neoadjuvant therapy	1.54 (0.99–2.38)	0.068	—	—	—	—	—	—
ypT	2.68 (0.38–18.70)	0.011	1.13 (0.01–180.12)	0.593	12.74 (0.91–177.51)	0.013	1.01 (0.28–3.65)	0.962
ypN	8.44 (4.64–15.32)	<0.001	8.49 (3.29–21.92)	<0.001	6.68 (2.46–18.15)	<0.001	20.62 (4.06–104.77)	<0.001
TRG	1.24 (0.63–2.45)	0.257	3.17 (0.84–11.97)	0.083	0.59 (0.17–2.03)	0.148	1.53 (0.36–6.52)	0.723
Lymph nodes removed	0.59 (0.45–0.79)	<0.001	0.59 (0.35–1.01)	0.052	0.62 (0.39–0.97)	0.035	0.28 (0.11–0.74)	0.01
Surgical approach	0.51 (0.24–1.09)	0.095	1.28 (0.21–7.64)	0.519	0.12 (0.02–0.96)	0.127	1.13 (0.49–2.56)	0.164
Anastomosis site	2.19 (0.43–11.17)	0.159	2.69 (0.39–18.73)	0.49	2.06 (0.21–4.53)	0.013	—	—
Adjuvant therapy	1.48 (1.06–2.06)	<0.001	1.56 (0.86–2.84)	0.006	1.36 (0.79–2.31)	0.3	1.60 (0.67–3.83)	0.336

Emdash indicates that the representative cannot be calculated. Total: 658 patients.

TRG, tumor regression grade; NCT, neoadjuvant chemotherapy; NICT, neoadjuvant chemoimmunotherapy; NCRT, neoadjuvant chemoradiotherapy; NICRT, neoadjuvant chemoradiotherapy plus immunotherapy.

## Discussion

To date, previous studies have primarily focused on postoperative survival in neoadjuvant-treated EC patients and the prognostic impact of different neoadjuvant combination regimens (5–7, 11, 17, 18). While neoadjuvant therapy provides improved survival for LAEC patients, nearly 50% experience disease progression during long-term follow-up (5–7); moreover, NCT and NICT patients predominantly manifest locoregional recurrence (7, 19, 20), while NCRT/NICRT patients mainly develop distant metastasis (21–23).

This study is among the limited research primarily investigating specific sites of LNR in EC patients after neoadjuvant therapy. LNR region nomenclature adhered to JES definitions (16), with analysis focusing on LNR patterns stratified by primary tumor location, neoadjuvant regimen (NCT, NICT, and NCRT/NICRT), and adjuvant therapy strategies. At our institution, the pCR rate reached 26.0% in neoadjuvant-treated EC patients, with patients receiving NCRT/NICRT achieving a pCR rate as high as 46.5%. The most frequent recurrence sites for overall EC were No. 104, No. 106tb, and No. 109, with metastasis rates exceeding 5%. This aligns with prior studies reporting similar common recurrence regions in thoracic EC patients undergoing radical esophagectomy (12, 24); however, as all patients in this study received neoadjuvant therapy, LNR rates in these identical regions were significantly reduced compared to historical data. In upper/mid-thoracic EC, LNR frequently involved supraclavicular and upper mediastinal regions, while abdominal LNR incidence remained low, consistent with JCOG0502 and the study of Zhao et al. (12, 25). However, in our study, supraclavicular LNR rates also exceeded 5% in lower thoracic and GEJ EC patients, a pattern similarly reported by Zhao et al. (13).

For different neoadjuvant regimens, the study by Wu et al. (12) and the 2-year follow-up of the Neoadjuvant Immunotherapy Chemotherapy for Local advanced Esophageal cancer (NICE) trial (20) indicated that mediastinal regions were common LNR sites in EC patients treated with NCT or NICT. Although both studies had limited sample sizes and few mediastinal recurrence events, our findings align with theirs. Additionally, we identified No. 104 as another high-risk LNR region. However, in our NCRT/NICRT cohort, No. 104 recurrence exceeded 5%, potentially attributable to the predominance of NICRT patients (83%) enrolled predominantly from another prospective clinical trial at our center (26). The radiation protocol employed a 30-Gy total dose with involved field irradiation; the biologically effective dose (BED) was approximately 40 Gy, featuring smaller target volumes and a lower biologically effective dose than conventional NCRT. This approach may have inadequately cleared occult lymph node micrometastases outside the radiation field. The overall anastomotic recurrence rate was 3.0%, significantly lower than historical rates, although potentially influenced by shorter follow-up.

While the National Comprehensive Cancer Network (NCCN) guidelines do not recommend adjuvant therapy for R0-resected ESCC post-esophagectomy, retrospective analyses and prospective studies indicate that adjuvant radiotherapy reduces locoregional recurrence and improves survival (27–29). Currently, no unified standard exists for postoperative radiotherapy target delineation in EC (30). Internationally accepted clinical target volumes (CTVs) primarily encompass bilateral supraclavicular/upper mediastinal regions (No. 104, No. 105, No. 106, and No. 107) (31), with lower thoracic EC (≥3 metastatic nodes) requiring additional abdominal coverage (No. 1, No. 2, No. 3, and No. 7) (32). In our study, adjuvant radiotherapy demonstrated significantly lower LNR rates within CTV-covered areas compared to adjuvant chemotherapy/chemoimmunotherapy. However, No. 109—located outside CTV boundaries—showed relatively high recurrence rates. Concurrently, No. 104 recurrence exceeded 10%, potentially attributable to the following: omission of No. 104 dissection to reduce surgical difficulty, suboptimal radiation dose, and residual occult nodal metastases. Further stepwise stratified analyses are warranted to elucidate these mechanisms.

Advancing T and/or N stages significantly elevates nodal metastasis probability in EC. The esophagus’ dense lymphatic network facilitates diverse metastasis patterns—regional, skip, or distant metastases (4). Higher lymph node dissection counts correlate with enhanced locoregional control (33–35). Pathological nodal status remains a validated prognostic marker—multicenter data confirm escalating recurrence risk with worsening nodal pathology in surgery-only cohorts (36). Our study established ypT/N stages and lymph node yield as independent LNR risk factors, suggesting that more extensive lymphadenectomy may reduce post-neoadjuvant LNR risk.

This study has several limitations. First, although the sample size allowed adjustment for multiple confounding factors, the single-center retrospective design, variations in perioperative treatment strategies, and the inclusion of both SCC and adenocarcinoma populations represent potential sources of bias. Enlarging the adenocarcinoma subgroup in future studies would help reduce potential bias in the reported LNR rate. Second, due to incomplete data availability, clinicopathological variables were analyzed retrospectively, and some features showed an imbalanced distribution, which may compromise the reliability of subgroup analyses. Finally, the diversity in neoadjuvant and adjuvant treatment regimens may have influenced LNR outcomes. Therefore, future trials should conduct stepwise analyses to assess whether LNR risk-adapted neoadjuvant/adjuvant strategies improve short-term RFS.

## Conclusions

No. 101, No. 106tb, and No. 104 represent common recurrence sites for both neoadjuvant-treated EC and those with upper/mid-thoracic tumors. Vigilance for No. 104 metastasis is critical in lower thoracic/GEJ EC or NCRT/NICRT-treated patients. Adjuvant radiotherapy effectively reduces mediastinal nodal recurrence.

Furthermore, a more thorough lymphadenectomy and the administration of adjuvant therapy following neoadjuvant treatment are key factors associated with reduced risk of LNR. Conversely, advanced ypT and/or ypN stages portend elevated LNR susceptibility.

## Data Availability

The original contributions presented in the study are included in the article/supplementary material. Further inquiries can be directed to the corresponding authors.
